# Understanding glioblastoma stromal barriers against NK cell attack using tri-culture 3D spheroid model^☆^

**DOI:** 10.1016/j.heliyon.2024.e24808

**Published:** 2024-01-20

**Authors:** Marcel Alexander Heinrich, Ngoc-Tien Huynh, Lena Heinrich, Jai Prakash

**Affiliations:** Department of Advanced Organ Bioengineering & Therapeutics, Engineered Therapeutics Section, Technical Medical Centre, University of Twente, 7500AE, Enschede, the Netherlands

**Keywords:** 3D GBM spheroids, Glioblastoma multiforme, Astrocytes, Microglia, Natural-killer cells, Chemotherapy, Immunotherapy

## Abstract

Glioblastoma multiforme (GBM), a highly aggressive tumor type with a dismal survival rate, has a poor outcome which is at least partly attributed to the crosstalk between cancer cells and cells from the tumor microenvironment such as astrocytes and microglia. We aimed to decipher the effect of these cells on GBM progression and on cell-based therapies using 3D co-cultures. Co-culturing of glioblastoma cells with patient-derived astrocytes or microglia or both formed dense and heterogeneous spheroids. Both, astrocytes and microglia, enhanced the spheroid growth rate and formed a physical barrier for macromolecules penetration, while only astrocytes enhanced the migration. Interestingly bi-/tri-cultured spheroids showed significant resistance against NK-92 cells, likely attributed to dense stroma and induced expression of immunosuppressive genes such as IDO1 or PTGES2. Altogether, our novel 3D GBM spheroid model recapitulates the cell-to-cell interactions of human glioblastoma and can serve as a suitable platform for evaluating cancer therapeutics.

## 1 Introduction

Glioblastoma multiforme (GBM) is the most prevalent malignant brain tumor with a devastating prognosis for patients [[1], [2], [3]]. Despite tremendous treatment efforts, patients diagnosed with GBM usually have an average overall survival time of only 15 months, including patients who underwent surgical resection or treatment with chemotherapy [1,2]. One of the reasons for this low survival rate is the highly aggressive and invasive behavior of GBM, which drastically limits the efficacy of surgical resection of the tumor, as well as high resistance towards chemo- and radio-therapy [4,5]. Temozolomide is the first choice chemotherapeutic agent for the treatment of GBM; however patients develop resistance against this drug [6]. Combination of temozolomide with tumor-treating fields (locoregionally delivered antimitotic treatment) has been shown to induce the median overall survival to 20.5 months compared to the standard chemotherapy (15.6 months) [7]. In recent years it has been shown that a variety of non-malignant cells in the GBM stroma play a significant role in this aggressive behavior of GBM [3,8,9]. In particular, microglia and astrocytes have recently shown to increase the invasiveness and aggressiveness of GBM as well as resistance towards chemotherapy [[9], [10], [11], [12], [13], [14], [15], [16]]. However, there is a lack of understanding how these cells contribute to the formation of physical and biological barriers of GBM stroma.

In contrast to animal models either lacking immune system (xenografts) or human species (syngeneic models), 3D in vitro models allow for inclusion of both immune cells and human cells in a cost-effective manner [[17], [18], [19], [20]]. They also allow for studying cellular interactions, resistance mechanisms and other biological features in depth in contrast of in vivo studies. We have earlier developed a 3D bioprinted GBM model to study the interaction between GBM and the healthy host immune environment in greater detail [21]. While such 3D bioprinted GBM models offer a great advantage on constructing 3D models with well-defined architecture [[21], [22], [23]], 3D spheroids are still one of the most promising in vitro platforms for high-throughput drug screening when focusing on replicating a tumor region alone [[24], [25], [26], [27]]. Some studies have recently attempted to recapitulate GBM characteristics using 3D spheroids [[28], [29], [30]]. They either generated mono-cultured 3D spheroids from GBM cell lines only [28,29] or co-cultured with astrocytes [30].

Although all of these studies were able to generate 3D in vitro GBM models that recapitulate different characteristics of GBM such as a high growth rate and invasion or include specific GBM components such as astrocytes, these models are still limited to the cellular number and complexity. Furthermore, they lack a cell type that has been shown to play a crucial role in GBM progression and treatment resistance i.e. microglia. In particular, the increasing focus on effective immunotherapies for cancer treatment requires reproducible in vitro models that include such crucial immune components [25,26].

In this study, we investigated the effect of astrocytes and microglia on different GBM characteristics such as proliferation, invasion and resistance to different therapies in the form of 3D GBM spheroids by incorporating primary patient-derived astrocytes and established cell lines of microglia and glioblastoma cells. We demonstrated that the incorporation of both, astrocytes and microglia, significantly increased the growth of the tricultured spheroids as well as demonstrated how astrocytes play crucial roles in the invasion of GBM. Furthermore astrocytes and microglia contribute to a dense physical barrier protecting GBM from the infiltration of macromolecules or cells. Novel therapeutics are being developed that aim to specifically modulate the GBM microenvironment, including immunotherapy, cell-based immunotherapies, or ECM-remodeling therapeutics [[31], [32], [33], [34], [35], [36]]. We found that co-culture with stromal cells increased the resistance to cytotoxicity caused by natural killer (NK) cells. We believe that our 3D in vitro model will to advance the understanding of complex cellular interactions and barriers formation in the GBM stroma as well as serve as a simple and reproducible platform for advanced therapeutic testing.

## 2 Results

### 2.1 Formation of 3D GBM spheroids by co-culturing astrocyt

**Fig. 1:**
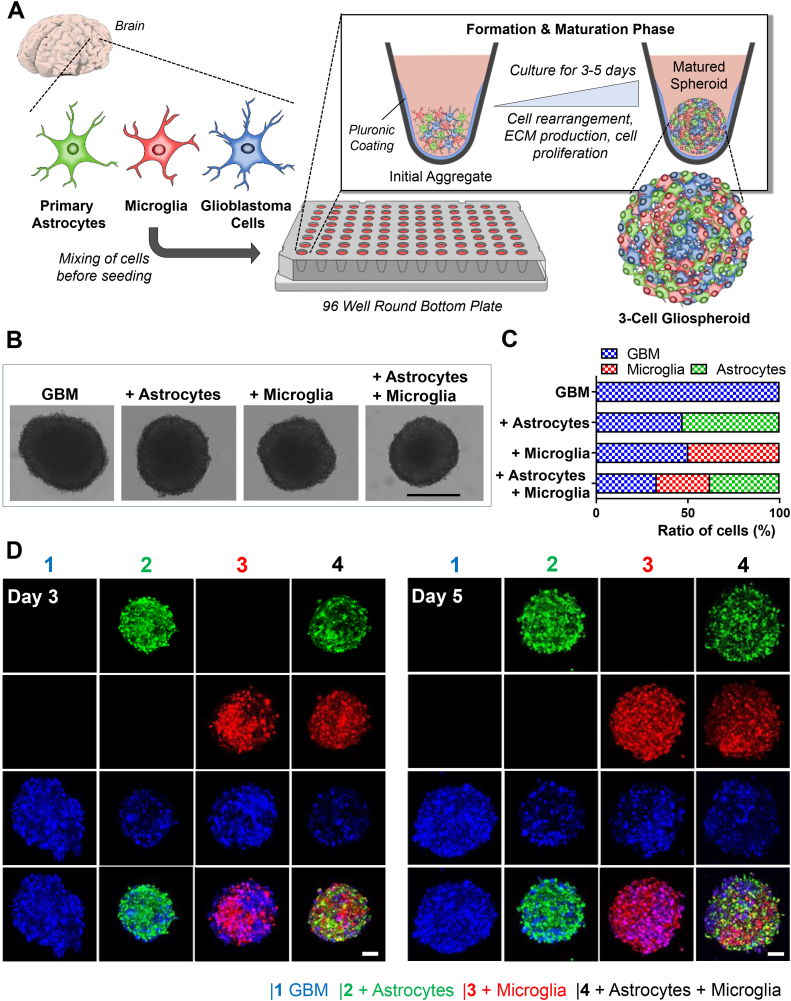
Composition of 3D GBM spheroids consisting of GBM cells, astrocytes and microglia. A) Schematic representation of the process to generate novel 3D GBM spheroids consisting of primary astrocytes, HMC3 microglia cells and U87MG glioblastoma cells describing the co-culture of the cells in pluronic-coated U-bottom 96 well plates for 3–5 days to form stable and matured 3D GBM spheroids. B) Microscopic images of the formed 3D GBM spheroids on day 3 post-seeding, scale = 500 μm, C) Ratio of cells in 3D GBM spheroids (in %) on day 3 after seeding, n = 3. D) confocal microscopy images of CellTracker™-labeled 3D GBM spheroids on day 3 and 5 post-seeding, scale = 100 μm.

With the aim to understand the role of different stromal cells (astrocytes and microglial) on the cellular interactions with tumor cells, we developed 3D spheroids by co-culturing either human primary astrocytes or human microglia (HMC3 cell line) or both together with human GBM cells (U87MG) in a systematic manner. The strategy we present here is based on the co-culture of these cells in a standard U-bottom shaped 96-well plate coated with pluronic F-127 to avoid cell adhesion (Fig. 1A). After 3 days of undisturbed culture under normal culture conditions, we observed that 3D GBM spheroids alone as well as the combinations of astrocytes/GBM (ratio 60:40), microglia/GBM (ratio 50:50) and all three cell types (astrocytes/microglia/GBM, ratio 40:30:30) form stable spheroids (Fig. 1B). We included a higher amount of astrocytes in the spheroids compared to microglia and GBM to replicate the abundance of astrocytes in the brain and better mimic the realistic situation found in vivo [12,37].

We first investigated the cellular composition and arrangement in our novel developed spheroid platform. Hereby, we labeled the cells using fluorescent CellTrackers™ prior to seeding to track astrocytes in green, microglia in red and GBM in blue, respectively. We found that after 3 days of culture, hence at the time of stable formation, the spheroids still displayed on average the ratios of cell that we added during the seeding of the spheroids indicating that in the period of 3 days, the spheroids primarily assemble and cells arrange, with reduced proliferation (Fig. 1C). In detail the combination of astrocytes/GBM displayed a ratio of 52.8%/47.15 (±4.44%), the combination of microglia/GBM a ratio of 49%/51% (±19%) and the combination of all three cell types a ratio of 37% (±4.1%), 29.02 (±10%) and 33% (±7.1%) for astrocytes, microglia and GBM, respectively.

Our confocal microscopy data showed that after 3 days of culture, the cells formed well matured 3D GBM spheroids without any clear clusters or aggregation of a specific cell type in the combination of different cells (Fig. 1D). Furthermore, no specific alterations in the cellular rearrangement could be observed after 2 additional days of culture indicating that the spvong time.

### 2.2 3D GBM spheroids present viable and dense cellular constructs

**Fig. 2:**
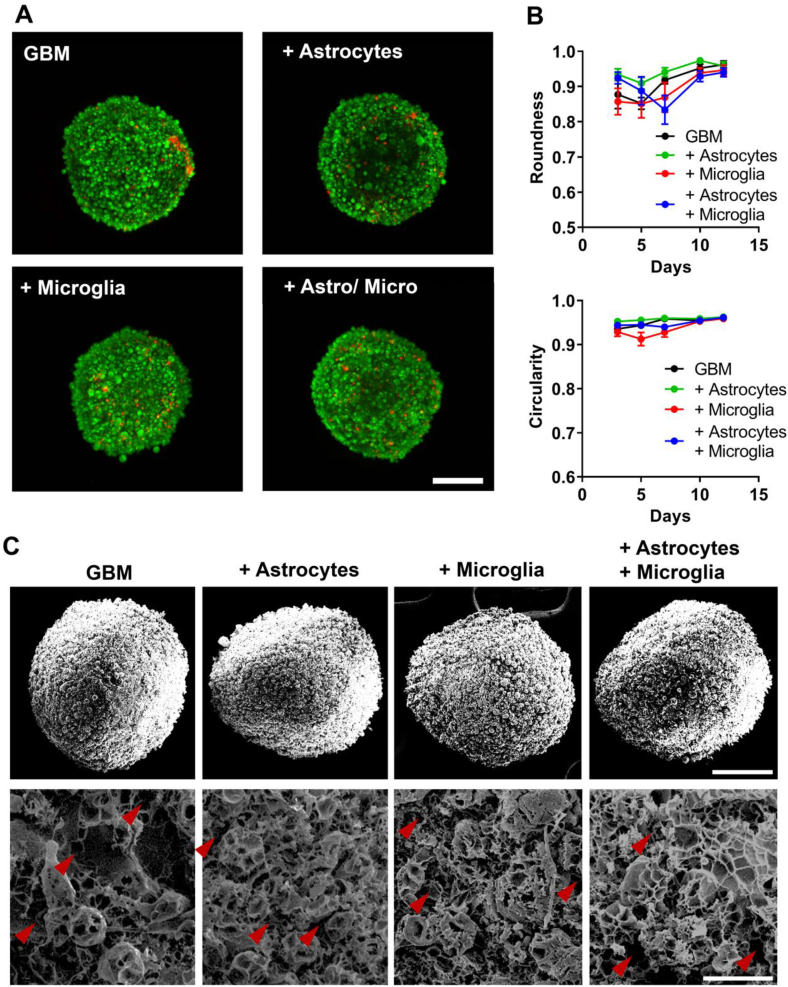
Effect of astrocytes and microglia on the shape and compactness. A) LIVE (green)/DEAD (red) staining in optically cleared 3D GBM spheroids using confocal microscopy on day 5 (scale = 200 μm). B) Roundness of 3D GBM spheroids followed for a duration of 12 days based on microscopic images (top) and circularity of 3D GBM spheroids followed for a duration of 12 days based on microscopic images (bottom). C) Scanning electron microscopy images of lyophilized 3D GBM spheroids on day 5 post-seeding at (top) 120× magnification and (bottom) 500× magnification. Red arrows indicate clear gaps between the cells (scale (top) = 200 μm, scale (bottom) = 25 μm). (For interpretation of the references to colour in this figure legend, the reader is referred to the Web version of this article.)

Studies have shown that GBM represents a dense tumor mass and contains various stromal cells. To understand whether astrocytes and/or microglials contribute to the formation of dense GBM stroma, we characterized the spheroids with mono-/di-/tri-cultures, as explained in Fig. 1, for their viability, compactness, and cellular density as well as morphology. One of the most characteristic features of spheroid cultures is arguably the capability of achieving a high density and compactness, which is mainly based on the cell rearrangement and production of their own ECM. As aforementioned, we avoided the need for external matrix components to stimulate cell aggregation as well as to improve the shape and compactness of spheroids. Before closer investigating these characteristics of our spheroids, we examined the overall viability of cells after culture for 5 days. We found that all spheroids display a high viability, displaying only a few spots of dead cells, which seem randomly distributed, indicating that after 5 days no hypoxic or necrotic core was formed (Fig. 2A and Supplementary Fig. S2). After confirming viability, we determined if our novel 3D GBM spheroids indeed show a high compactness by investigating the shape of the spheroids in terms of circularity and roundness. We found that all spheroids display a very high circularity of >0.9/1, where 1 represents a perfect circle as well as a high roundness of >0.8/1 (Fig. 2B), indicating a high compactness of the 3D GBM spheroids. Interestingly we also observed that once spheroids are cultured for more than 7 days, the circularity and roundness drastically increase and achieve values for circularity and roundness of around 0.95 for all cultured 3D GBM spheroids, which shows that spheroids stay compact and stable for a longer culture duration.

To further confirm the shape and compactness of our novel 3D GBM spheroids, we examined their shape and surface structure using scanning electron microscopy after lyophilization after culture for 5 days. We found that all spheroids display a highly spheroidal 3D shape under SEM (Fig. 2C). Furthermore, we could confirm the size differences between the different spheroids as mentioned above. Interestingly, we also observed that all spheroids have a very homogenous surface structure without any significant differences among the whole surface. Further magnifying the surface of the spheroids, however, revealed certain differences among the different spheroid cultures. 3D GBM spheroids displayed larger gaps in between the cells, indicating bigger pores between the cells and overall less density of the spheroids. The combination of the different cells (astrocytes/GBM, Microglia/GBM and astrocytes/microglia/GBM) display similar surface structures, all with smaller pore sizes compared to 3D GBM spheroids alone, indicating a higher compactness, with similar protrusion indicating a highly invasive profile of all four spheroids.

### 2.3 Astrocytes and microglia alter the cellular arrangement within 3D GBM spheroids and contribute to a dense physical barrier

**Fig. 3:**
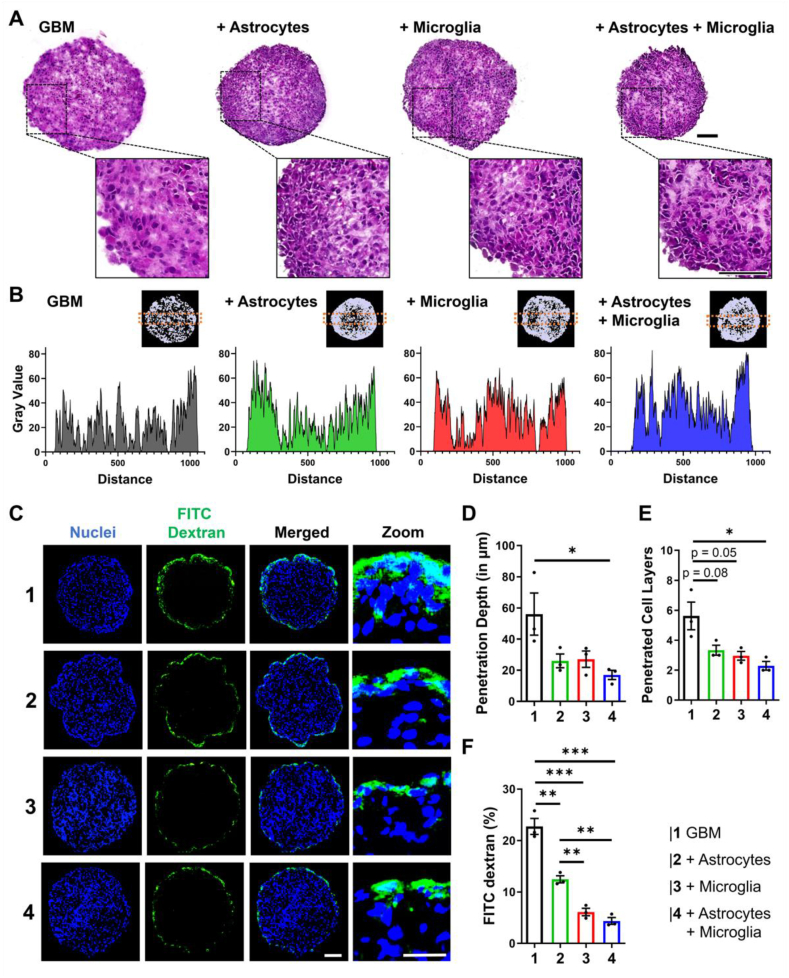
Astrocytes and microglia alter spheroid morphology and contribute to physical barrier formation. A) Hematoxylin/Eosin staining of cryo-sectioned 3D GBM spheroids on day 5 post-seeding for (top) the whole spheroids and (bottom) zoom-in (scale = 100 μm) B) Spatial distribution of cells based on nuclear density within 3D GBM spheroids based on HE staining depicted as intensity over distance for a defined area in 3D GBM spheroids (highlighted by orange box in miniature image of nuclei). C) Penetration of 150 kDa molecular weight FITC-Dextran (green) into 3D GBM spheroids. Counterstained with DAPI for nuclei (blue) (scale = 100 μm; zoom scale = 25 μm). D-F) Quantification of the penetration of FITC-Dextran based on the (D) depth in μm, (E) the penetrated amount of cell layers and (F) the overall based on ratio of the surface area of FITC-Dextran to nuclei, n = 3. Data represent mean ± standard error of the mean. Statistical analysis was performed by two-tailed Student’s *t*-test. *p < 0.05, **p < 0.01, ***p < 0.001. (For interpretation of the references to colour in this figure legend, the reader is referred to the Web version of this article.)

While the aforementioned shape and surface structure of the GBM spheroid give some indication on the cellular arrangement, we were interested in investigating the morphology within the 3D GBM spheroids in greater detail. We performed a hematoxylin/eosin (H/E) staining to better understand the general composition of the 3D GBM spheroids (Fig. 3A). Interestingly, we found that especially 3D GBM spheroids display less packed cells. This supports the larger pore size found while investigating the surface structure with SEM, indicating that more gaps between the cells are related to the different cell size and their packing. The protrusions on the surface of all spheroids might be due to the rapid growth of tumor cells which tend to grow outward and form finger-like shape in real tumors. Similar to the SEM results, the spheroid combinations of astrocytes/GBM, microglia/GBM and astrocyte/microglia/GBM display very similar morphologies with areas of more gaps as well as areas with cells close packed together. Again, this falls in line with the observation from the SEM imaging, depicting higher compactness GBM. Directly comparing the position of nuclei within the spheroids confirmed the more loose structure of 3D GBM spheroids, while the combinations clearly dispatch a high cell density at the outer rim of spheroids (Fig. 3B and Supplementary Fig. S2). Intriguinly, the combination of GBM/astrocytes/microglia displayed the highest density with a maximum gray value of 80 compared to 65, 65 and 70 for GBM, GBM/astrocyte and GBM/microglia, respectively. Furthermore, it can be seen that GBM/microglia and GBM/astrocyte/microglia also display higher cellular density within the spheroids, while cell in GBM/astrocyte spheroids are more located towards the outer part, which demonstrates an overall higher cellular of spheroids containing GBM and microglia or the combination of all 3 cell types.

Next, based on the differences observed for the cellular density, we wanted to investigate the formation of a physical barrier in our 3D GBM spheroids by determining the resistance towards the general penetration of macromolecules into the GBM microenvironment. For this, we evaluated the penetration of FITC-Dextran of 150 kDa molecular weight based on the penetration depth, the amount of cell layers penetrated by the dye and the surface area of FITC-Dextran penetrated into the spheroid (Fig. 3C–F). In line with the previously shown loose structure of 3D GBM spheroids, we found that 3D GBM spheroids display a high penetration of macromolecules with a depth of 56 ± 23.42 μm, 5.6 ± 1.59 cell layers and an overall surface area of 22.7 ± 2.6 %, respectively.

Intriguinly, the addition of astrocytes, microglia and the combination of astrocytes/microglia significantly reduces the penetration of FITC-Dextran. In particular, the combination of GBM, astrocytes and microglia displays the lowest penetration with a penetration depth of 17 ± 5.12 μm, a cell layer penetration of 2.29 ± 0.5 layers and a total surface area of 4.36 ± 1.15 %, displaying the clear influence of astrocytes and microglia in this co-culture, which is also supported by the higher density presented earlier.

### 2.4 Astrocytes and microglia stimulate proliferation and invasion of 3D spheroids

**Fig. 4:**
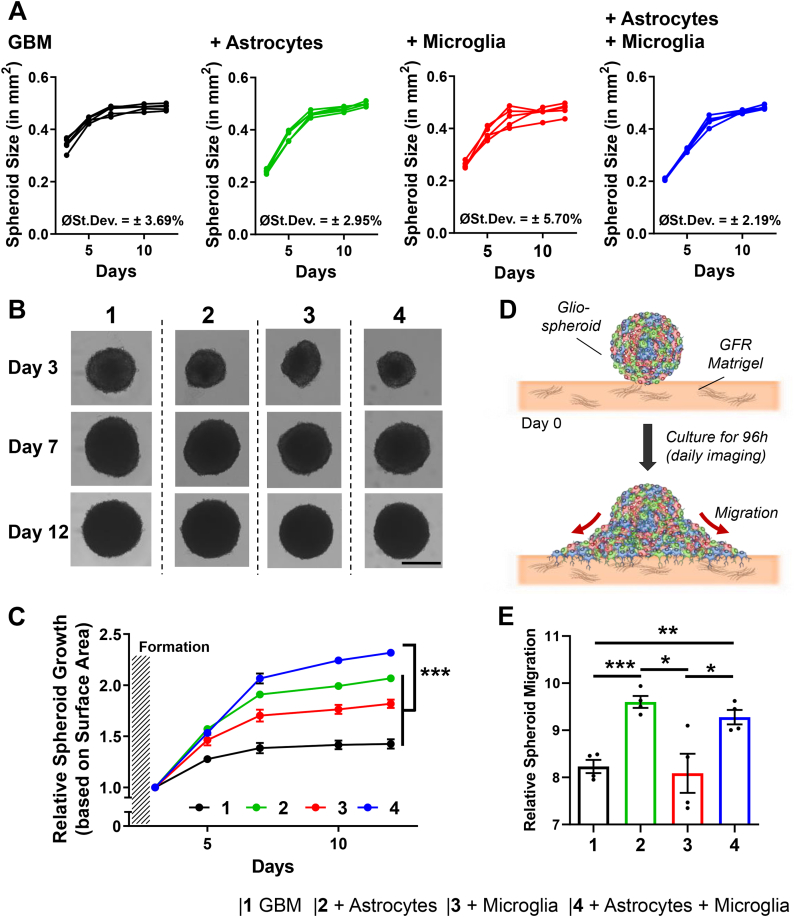
Effect of astrocytes and microglia on GBM spheroid growth rate and migration. A) Individual growth profile for 3D GBM spheroids for 5 independent experiments followed for a total duration of 12 days. Highlighted is the combined standard deviation for the total culture duration. B) Microscopic images of 3D GBM spheroids on day 3, day 7 and day 12 post-seeding. Scale = 500 μm. C) Growth rate of 3D GBM spheroids standardized to day 3 post-seeding and followed for a total duration of 12 days post-seeding, n = 5, significance is displayed for the final day of culture (day 12) comparing the triculture vs. GBM alone, GBM + astrocytes and GBM + microglia. D) Schematic representation of the experimental setup to study the migration of 3D GBM spheroids on top of a matrigel layer (GFR = growth factor reduced). E) Relative spheroid migration after 4 days based on the calculated invaded surface area and standardized to day 0, n = 4. Data represent mean ± standard error of the mean. Statistical analysis was performed by two-tailed Student’s *t*-test. *p < 0.05, **p < 0.01, ***p < 0.001.

After we have shown that the novel 3D GBM spheroids display spheroid-characteristic compactness and density, we were interested to demonstrate that our novel 3D GBM spheroids can be used to study GBM specific characteristics in greater detail. Here, we evaluated the effects astrocytes and microglia on the growth as well as on the migratory properties of the spheroids in more detail. We followed the growth of the spheroids for a total duration of 12 days displaying a clear growth profile of the different spheroid cultures. First, We found that based on 5 independent experiments, all spheroids display a very similar growth profile with a low averaged standard deviation (SD, calculated summarizing all culture days) ranging from ±2.19% for astrocytes/microglia/GBM to a maximum of ±5.70% for microglia/GBM. This low variation among the independent experiments confirms a high reproducibility of the GBM spheroid culture (Fig. 4A). Comparing the growth rate of the conditions, we observed that all GBM + stroma combinations display an overall higher growth rate compared to the single GBM culture, which only achieves an average growth rate of 1.42 ± 0.10, while the combination of all 3 cells, astrocytes/microglia/GBM, displayed significant higher growth rate compared to all other groups with a factor of 2.31 ± 0.05 compared to 2.06 ± 0.06 for astrocytes/GBM and 1.81 ± 0.09 for microglia/GBM (Fig. 4B and C**)**. This clearly demonstrates the importance of both, astrocytes and microglia, on the growth of GBM, where astrocytes seem to have a slightly higher impact on the growth compared to microglia.

GBM is also known for its high migrative properties into the healthy brain tissue [11]. To determine if the addition of astrocytes and microglia also has significant influence on the migration of the GBM in general, we determine the migration of our 3D GBM spheroids onto a 3D layer of growth-factor reduced (GFR) Matrigel (Fig. 4D and Supplementary Fig. S3). After tracking the migration for 4 days we observed that, despite an overall high migration of GBM in general (8.23 ± 0.28, value represents relative migration standardized to the start of the experiment), demonstrating the high invasive profile of glioblastoma cells, the combination of astrocytes/GBM and astrocytes/microglia/GBM displayed a significantly higher migration compared to the other spheroids with 9.60 ± 0.25 and 9.27 ± 0.30, respectively (Fig. 4E). This confirms the advantage of the novel 3D GBM spheroids compared to the single cultures as well as highlighting the potential importance of astrocytes in the migration process.

### 2.5 Astrocytes and microglia alter gene profiles of 3D GBM spheroids

After investigating the influence of astrocytes and microglia on the functional characteristics, we wanted to determine how the addition of astrocytes and/or microglia changes the overall genetic profile of the 3D GBM spheroids. We hereby focused on genes relevant to fundamental processes and interactions within GBM including the crosstalk between GBM cells and astrocytes/microglia, angiogenesis, matrix production & remodeling, GBM cell invasion, general markers of inflammation, the presence of cancer stem cells as well as the suppression of immune cells.

**Fig. 5:**
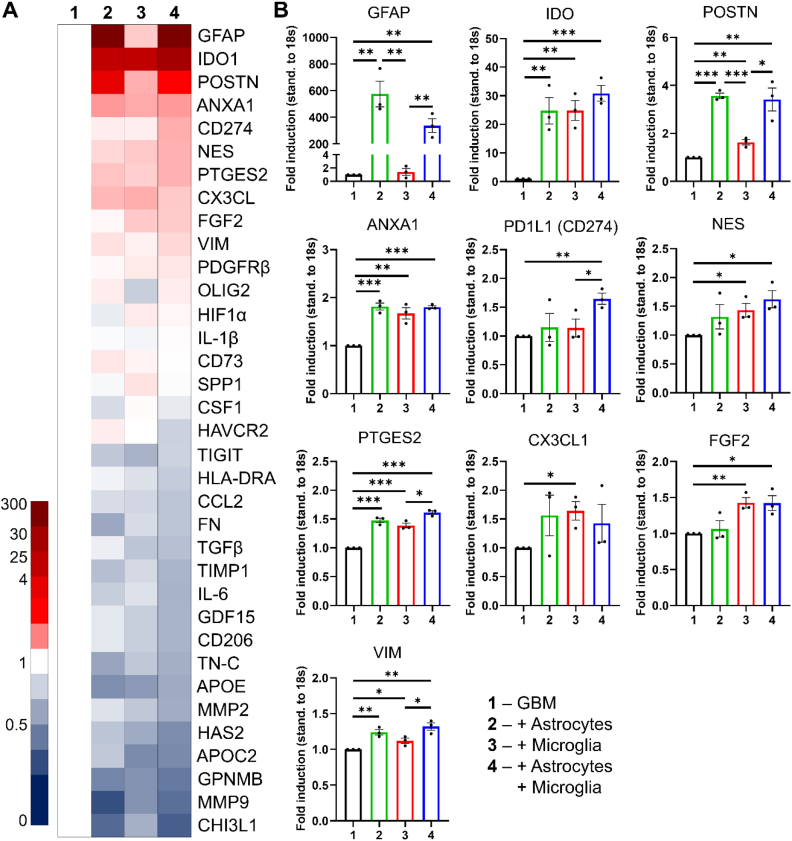
Gene expression profile of 3D GBM spheroids. A) Heat map of genes expressed in 3D GBM spheroids standardized to the expression in spheroids consisting solely of glioblastoma cells. B) Individual expression profiles highlighting the top 10 genes displaying the highest upregulation in the tricultured spheroids compared to 3D GBM spheroids, n = 3. Data present mean ± standard error of the mean. Statistical analysis was performed by two-tailed Student’s *t*-test. *p < 0.05, **p < 0.01, ***p < 0.001.

We found that the addition of either astrocytes, macrophages or both resulted in an increased expression in several genes related to crucial processes within GBM (Fig. 5 and Supplementary Fig. S4). For instance, several genes related to the crosstalk and the activation of the GBM stroma were highly upregulated in the different co-culture, such as GFAP, POSTN, ANXA1, CX3CL or PDGFRβ (Fig. 5A and B; full list of gene names and abbreviations in Supplementary Table S1). Remarkably, GFAP a well-known marker for Glioma-associated astrocytes (GAAs), strongly upregulated in our 3D GBM spheroids by 575-fold (bi-culture with GBM cells) and 337-fold (tri-culture), confirming the successful differentiation towards a GAA-like state [13]. Interestingly, POSTN, a known ECM protein involved in the crosstalk between GBM and microglia [38], displays and even higher upregulation in the GBM – astrocyte co-culture and the triculture with 3.56- and 3.41-fold, respectively, compared to the GBM/microglia co-culture with 1.63-fold, indicating that astrocytes might even play a more important role in the expression of POSTN. Interestingly, ANXA1, a marker relevant to the GBM-GAA crosstalk, is similarly upregulated in GBM – microglia co-culture or the triculture (1.81-, 1.67- and 1.8-fold, respectively), indicating that ANXA1 is a marker for a general activated GBM stroma [39]. Interestingly, markers related to cancer stem cells or angiogenesis are mainly upregulated in the presence of microglia. For instance, NES displayed a significant upregulation in GBM microglia and the triculture with 1.43 ± 0.2 and 1.62 ± 0.25 times accordingly. This upregulation of NES indicates the presence of glioma stem cells in the spheroid culture [40]. Similarly, we observed an increased microglia-mediated expression of FGF2 indicating angiogenic drive in the spheroids, which is known to be particularly maintained by glioma associated microglia, indicating the presence of this activated microglia type in the spheroids [41].

Remarkably, several genes related to immune suppression are found to be upregulated in the different co-cultures. We found that especially the expression of IDO1 and PTGES2 are significantly upregulated upon the introduction of astrocytes or microglia. In the triculture, IDO1 displayed an upregulation of 30.85 fold, compared to around 24 fold for either astrocyte or microglia co-cultures, indicating a synergistic effect. Similarly, PTGES2 displayed the highest expression in the tricultre with 1.6 fold upregulation. It is known that PTGES2 and IDO1 have a combined effect on the suppression of NK-92 cells indicating that our 3D GBM spheroids have NK-92 suppressing properties [42]. Interestingly, PDL1 or also known as CD274, which plays a crucial role in the suppression of T and NK cell activity [43,44], is only significantly upregulated in the triculture containing GBM, astrocytes and microglia displaying a 1.64 fold upregulation, indicating the importance of both stroma cell types and the beneficiary effect of this triculture.

Besides genes that are upregulated in the different co-cultures, we found several genes downregulated when combining GBM with astrocytes or microglia, which indicates that these genres are primarily expressed by GBM cells and get diluted in the co-culture. Primarily, genes related to ECM remodeling (MMP9, MMP2, HAS2, TN-C, TIMP1, FN) or general GBM markers (CHI3L1, GPNMB) seem to be mainly expressed by GBM cells as displayed by a significant downregulation of these markers in the different co-cultures.

### 2.6 GBM stroma hinders cytotoxicity from NK-92 cells in 3D GBM spheroids

**Fig. 6:**
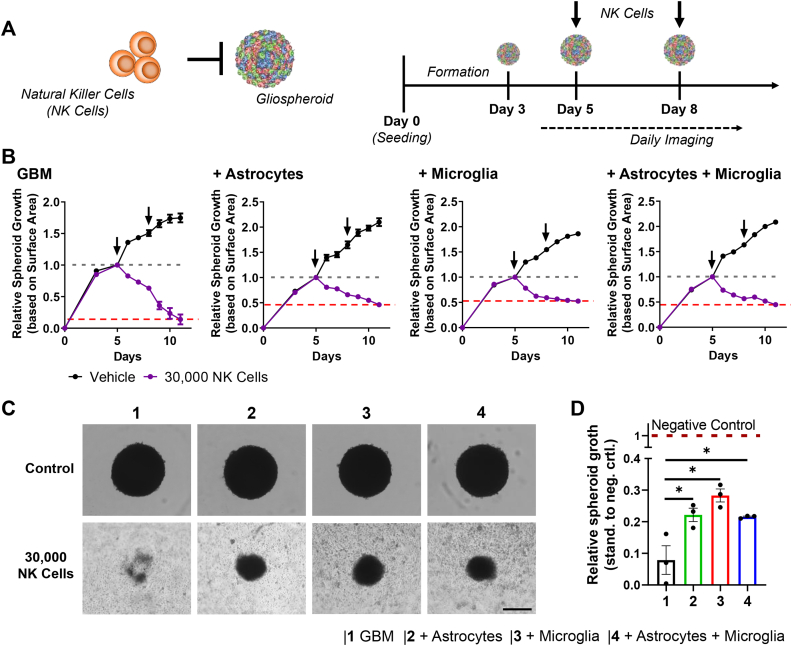
Effect of astrocytes and microglia on the cytotoxic effects of natural killer (NK) cells. A) Schematic representation of the experimental set-up for the co-culture of matured 3D GBM spheroids with NK-92 cells. B) Individual growth rate of 3D GBM spheroids after the incubation with vehicle or 30,000 NK-92 cells/well. Growth rate is standardized to day 5 (start of the co-culture) and followed for 6 consecutive days after start of the co-culture. Arrows indicate addition of NK-92 cells, n = 3. C) Microscopic images of 3D GBM spheroids on day 11 incubated with either vehicle (NK-92 cell medium) medium or 30,000 NK-92 cells (scale = 500 μm). D) Growth rate of 3D GBM spheroids on day 11. Each conditions was standardized to its representative control group treated with vehicle, n = 3. Statistical analysis was performed by two-tailed Student’s *t*-test comparing two specific groups. *p < 0.05.

In recent years, several studies revealed the importance of the tumor immune microenvironment in GBM progression and invasion [9,31]. In particular the suppression of the infiltrating immune cells such as macrophages, T cells and NK cells allows GBM to progress. Currently, there are engineered NK cell-based therapies in development as new therapeutics against GBM [33]. As we observed the addition of astrocytes and microglia increase the expression of genes related to immune cell suppression, especially NK cells (IDO1, PTGES2, PD1L1), we were interested to investigate if the addition of these cells can indeed inhibit the cytotoxic function of NK cells at a functional level. For this, we incubated the 3D GBM spheroids with either 15,000 or 30,000 NK-92 cells on day 5 after seeding and imaged the culture for 6 consecutive days, while refreshing the cells and medium on day 8 (Fig. 6A). We observed that 15,000 NK-92 cells as well as 30,000 NK-92 cells per well show a significant toxicity towards all cultures, including astrocytes, microglia and GBM cells (Fig. 6B–D and Supplementary Fig. S5). Interestingly, however, we found that 30,000 NK-92 cells are able to nearly completely eradicate spheroids consisting of GBM alone, while the combinations of GBM cells with astrocytes, microglia and astrocyte/microglia still remain at around 30% of their original size. Standardized to the growth of spheroids in control medium, it is shown that while all stroma-containing spheroids are more protected towards NK cell killing, microglia seemingly display a slight higher protective function compared to astrocytes (Fig. 6D). These results clearly indicate that both, astrocytes and microglia, play a significant role in the protection of GBM cells towards NK-92 cells and clearly inhibit the efficacy of NK-92 cells.

## 3 Discussion

In the last decades, astrocytes have often been overlooked on their importance in GBM progression and aggressiveness, however, in the recent years more studies focused on the importance of these cells on GBM proliferation and invasion [11,12,45]. It has been shown that in vivo astrocytes play a crucial role in these processes as well as greatly enhance the resistance towards different therapies such as chemo- or radiotherapies. Similarly, microglia have been shown to significantly support GBM progression as well as increase invasion of cancer cells [10,15,38]. However, so far a little is know about the synergistic effects of these two cell types to these crucial features of GBM.

To investigate the combined effect of astrocytes and microglia in a controlled fashion, this study presents a 3D GBM spheroid model that incorporates not only glioblastoma cells but also astrocytes and microglia to mimic the tumor-stroma interaction within the GBM microenvironment. Earlier patient-derived spheroids/organoids have been used to investigate patient-related heterogeneity induced by different mutations, different types of cells and ECM [46]. Although inclusion of heterogeneity is clinically relevant, this also poses a disadvantage to study the complex cell-to-cell interactions and effects of individual cells in a controlled manner due to unknown cell numbers and ratios as well as unknown cell phenotypes. Our 3D GBM spheroids were stable and successfully recapitulated the GBM-relevant features such as a glial-cell dependent growth, invasion and density as well as the formation of a physical barrier towards intercellular penetration. Furthermore, the addition of astrocytes and microglia resulted in several changes in the genetic profile indicating an activated GBM stroma as well as immunosuppressive conditions, which were subsequently confirmed by demonstrating the inhibition of NK-92 cell cytotoxicity.

Interestingly, we were able to not only confirm that astrocytes and microglia contribute to the progression of GBM demonstrated by the higher proliferation of the 3D GBM spheroids, but the especially the combination of GBM, astrocytes and microglia displayed the highest proliferation, demonstrating the synergistic effect. Interestingly, however, only astrocytes seem to be contributing to the migratory properties of GBM, demonstrated in the higher migration of spheroids containing astrocytes as well as in the higher expression of vimentin and periostin, which has recently been demonstrated to be directly related to GBM migration [45,47,48]. Moreover, Chen et al. earlier showed that the co-culture of microglia with GBM cells in 3D culture restricted invasion but enhanced proliferation, which is line of our findings [49].

One of the major features in our 3D GBM spheroids is the inhibition of NK-92 induced cell cytotoxicity, where it can be seen that both, astrocytes and microglia, significantly contribute to the suppression. Interestingly, studying the penetration of FITX-dextran, we observed significant differences in the formation of physical barrier, where the least penetration was observed in the triculture, followed by microglia and astrocytes bi-cultures. However, these differences were not visibile in the NK-92 cell killing assay, indicating that the suppression of NK-92 cells in our model might be based on the formation of a biological barrier, based on the expression of IDO1, PTGES2 and PDL1, rather than the formation of a physical barrier. Interestingly, IDO1 is known to be mainly expressed by microglia [50], however, we found significant upregulation in astrocytes co-cultures as well demonstrating their effect on immune suppression, which so far might not be fully understood.

Moreover, other genes related to immune suppression such as TGFβ, TIGIT or HAVCR2 seem to be primarily expressed by GBM cells, indicating an overall high suppression of the immune system by these cells, which is further increased by the stromal components [42].

Interestingly, PDL1 was only significantly expressed in the triculture and not in the bi-cultures, which is contradictory to literature indicating the primarily microglia are involved in the immune escape of GBM [51,52]. However, recent studies indicate that microglia and astrocytes display immune suppressive behaviour and that both cells are involved in the immune environnement of the brian [53,54], where astrocytes might even play a larger role than initially thought. Based on our observations the crosstalk between microglia and astrocytes could be a crucial element of the immune evasion in GBM and might be investigated more in future studies.

A crucial aspect about the microenvironment in GBM is the activated phenotype of the cells involved. In this study, we found several indications that the co-culture of astrocytes and/or microglia with GBM cells indeed resulted in an activated phenotype of these cells. GFAP, one of the major markers for glioma-associated astrocytes, is significantly upregulated in the co-cultures, indicating the presence of this phenotype [13]. 10.13039/100014337Furthermore, the enhanced proliferation and invasion indicates that astrocytes in their activated state support these characteristics which goes along with literature. Interestingly, we could not find clear markers for the activation of microglia in our model. While CD206 is a potential markers for such activation, we did not observe an upregulation of this markers [38]. Also other makers such as IL10 were not sufficiently expressed in the model [13,38]. However, the upregulation of FGF2 and NES indicates the presence of an activated microglia phenotype, as these processes such as angiogenesis and cancer stem cell formation are primarily driven by glioma-associated microglia rather than their quiescent phenotype [[55], [56], [57]]. However, future experiments are needed to fully confirm the presence of these specific phenotypes in our model.

## 4 Limitations of the study

Our model and the results are subject to several limitations. While we were able to display the contribution of astrocytes and microglia on the system as a whole, clearly identifying the individual geno- and pheno-typical changes for each cell type separately would require additional spheroid processing steps such as cell sorting or isolation. While such sorting can be achieved by fluorescence or magnetic activated cell sorting (FACS/MACS) combined with different staining techniques including immunofluorescent staining of surface markers, stable cell transfection to express fluorescent proteins or stable cell-tracking agents, such techniques were not successful in this study due to several reasons: i) the primary nature of astrocytes limited culture duration to 10 division cycles as recommended by the manufacturer which did not provide sufficient time to generate a stable transfection in these cells [58], ii) based on their often astrocytic origin, U87MG glioblastoma cells and astrocytes share highly similar markers for potential immunofluorescent stainings making it challenging to fully differentiate between the 2 cell types [59], iii) the used celltrackers were only able to provide sufficient fluorescence for a maximum of 5 days, which allowed for fluorescence microscopy on this day but not for sorting on later time points [60]. Future studies might be incorporating different more stable celltrackers or an astrocyte cell line which allows for stable transfection. Another limitations of the current study, is the use of microglia and glioblastoma cell lines. While this allowed for comparably simple culture conditions and longer culture durations, the use of primary human patient material would results in a more patient-relevant model, however, such primary cells are very limited in commercial availability. Future work might include the collaboration with a hospital that would allow for the use of materials from patients that, for instance, underwent surgical resection of the primary tumor. Lastly, the inclusion of other cell types, such as oligodendrocytes or neurons, would result in an even more patient-relevant model that might provide an even better model to evaluate novel cell or drug-based therapeutics [[61], [62], [63]], however, increased spheroid complexity would directly cause an increase in the culture and read-out complexity eventually.

## 5 Conclusion

In summary, the novel triculture in form of 3D GBM spheroids consisting of glioblastoma cells, astrocytes and microglia allowed for investigating GBM characteristics such a high invasion, progression as well as resistance to different therapeutic approaches. It was demonstrated how astrocytes overall promote the migratory behavior of GBM while microglia seem more involved in processes such as cancer stem cell niche formation or angiogenesis. Both cell types, however, play crucial roles in the progression of GBM as well as in the generation of a suppressive environment towards the immune system, as demonstrated by the decreased cytotoxicity of NK-92 cells. Altogether, the GBM promoting functions of the tumor stroma, in particular astrocytes and microglia could be demonstrated and with the addition of more GBM related cell types, novel crosstalks or interactions could be found, which might pave the way for new and more effective treatment options in the future. Due to the realistic biomimetic characteristics of our novel 3D GBM spheroids, they might serve as a model to evaluate these novel approaches, eventually leading to fast and more cost-effective drug development.

## 6 STAR methods

Key Resources Table.

**Table:** 

Reagent or Resource	Source	Identifier
Pluronic® F-127	Merck (Sigma-Aldrich)	Catalog #P2443 (CAS 9003-11-6)
Primary human astrocytes	ScienCell Research Laboratories	Catalog #1800
Human HMC3 microglia	American Type Culture Collection (ATCC)	Catalog #CRL-3304
Human U87MG glioblastoma cells	Gift from Prof. Schiffelers (UMC Utrecht), originally from ATCC	Catalog #HTB-14
Corning® Matrigel® (GFR) Basement Membrane Matrix, Phenol Red-free, LDEV-free	Corning	Catalog #356231
Fluorescein isothiocyanate (FITC) – dextran, average mol wt 150.000	Merck (Sigma-Aldrich)	Catalog #46946 (CAS 60842-46-8)
Temozolomide	Merck (Sigma-Aldrich)	Catalog #T2577 (CAS 85622-93-1)
NK92 Cells	DSMZ – German Collection of Microorganisms and Cell Cultures GmbH	Catalog #ACC488

### 6.1 Cell culture

Human U87MG glioblastoma-like cells were a kind gift from Raymond M. Schiffelers, University Medical Center Utrecht, The Netherlands. Human microglial HMC3 cells (#ATCC CRL-3304) were obtained from the American Type Culture Collection (ATCC, Manassas, VA, USA). Both, U87MG and HMC3 cells, were cultured in Dulbecco’s Modified Eagle Medium (DMEM, Lonza, Basel, Switzerland) supplemented with 10% fetal bovine serum (FBS, Sigma-Aldrich, St. Louis, MO, USA), 100 U/mL penicillin (Thermofisher Scientific, Waltham, MA, USA), 100 μg/ml streptomycin (Thermofisher Scientific) and 2 mM l-glutamine (Thermofisher Scientific). Primary human astrocytes (#1800) were obtained from ScienCell (Carlsbad, CA, USA) and cultured in astrocyte medium (AM) supplemented with 2% FBS, 100 U/mL penicillin, 100 μg/ml streptomycin and 1% astrocyte growth supplements. Natural killer (NK92) cells were purchased from the German collection of microorganisms and cell cultures GmbH (Leibniz-Institute DSMZ, Braunschweig, Germany) and cultured as recommended by the manufacturer. In brief, NK92 cells were cultured in alpha Minimum Essential Medium (αMEM, Thermofisher Scientific) with ribo- and deoxyribonucleosides containing 12.5% heat-inactivated FBS, 12.5% heat-inactivated horse serum (Sigma-Aldrich), 100 U/mL penicillin, 100 μg/ml streptomycin, 2 mM l-glutamine and 150 U/mL human recombinant interleukin-2 (PeproTech EC, Ltd., London, United Kingdom). All cell types were cultured in a humidified atmosphere of 5% CO2 at 37 °C. Adherent cultures were split at 80% confluence. NK92 cells were split at an average concentration of 600.000 cells/mL. All cells were kept between 2 and 20 passages, except primary astrocytes, which were cultured until maximum 6 passages.

### 6.2 GBM spheroid generation

3D GBM spheroids were generated based on the culture of cells on a low adherent surface. In brief, commercially available U-bottom 96 well plates (Cellstar®, Greiner Bio One, Kremsmünster, Austria) were incubated overnight with 1 w/v% Pluronic® F-127 (Sigma-Aldrich), washed twice with sterile Mili-Q water and air-dried before cell seeding. All cells were trypsinized using 0.25% trypsin-EDTA (Thermofisher Scientific, Waltham, MA, USA), counted and seeded at the following ratios (astrocytes: HMC3: U87MG, total amount of cells 6.000 cells/well): i) 100:0:0; ii) 0:100:0; iii) 0:0:100; iv) 60:0:40; v) 0:50:50; vi) 40:30:30. After seeding, the spheroids were cultured for at least 3 days without moving or disturbing the plate (unless mentioned otherwise). All spheroids were cultured under the same conditions using a mixture of full astrocyte culture medium and full DMEM culture medium (ratio 1:1).

### 6.3 Microscopic imaging of (pre-labeled) 3D GBM spheroids

3D GBM spheroids were prepared as previously described. Brightfield microscopy images were taken using an EVOS M5000 microscope (Thermofisher Scientific). For confocal microscopy, astrocytes, HMC3 and U87MG were fluorescently labeled using CellTracker® Green CMFDA, Orange CMRA and Blue CMAC (Thermofisher Scientific), respectively, before seeding. After 3 or 5 days of culture, the 3D GBM spheroids were washed with Dulbeccos’s Phosphate Buffered Saline (DPBS, Thermofisher Scientific), fixed with 4% formaldehyde (Sigma-Aldrich) and again washed twice with DPBS. 3D GBM spheroids were kept in DPBS and imaged using a Nikon A1 Confocal Laser Microscopy System (Nikon Instruments, Tokyo, Japan).

### 6.4 Cryosections of pre-labeled 3D GBM spheroids

Astrocytes, HMC3 and U87MG were labeled with CellTrackers® as previously described before seeding. 3D GBM spheroids were cultured for 3 days before being washed with DPBS, fixed with 4 v/v% formaldehyde, washed again twice with DPBS and embedded into Cryomatrix™ before being snap-frozen using isopentane. Embedded and frozen 3D GBM spheroids were cut into 6 μm thick cryosections, air-dried, rehydrated with PBS and immediately mounted with Fluoroshield™ with DAPI (Sigma Aldrich) before being imaged using Nanozoomer-RS (Hamamatsu Photonics, Hamamatsu, Japan). Scanned images were analyzed using Image J (Public, developed by Wayne Rasband (NIH)).

### 6.5 LIVE/DEAD (viability) Staining

3D GBM spheroids (day 5) were washed twice DPBS prior to staining with calcein AM (green, 1 μM) and ethidium homodimer-1 (EthD-1, red, 2 μM) in serum-free supplemented 50:50 medium. Spheroids were incubated for 20 min in volumes of 50 μL and were subsequently fixed with 4% formaldehyde (FA) for 15 min. The stained 3D GBM spheroids were then optically cleared with the clearing protocol adapted from Qi et al. without pH adjustment [64]: In brief, the 3D GBM spheroids were dehydrated in different solutions of tetrahydrofuran (THF) starting with 50% (in MiliQ) for 1 h, followed by 30 min incubation in 70%, 80%, and twice in 100% THF respectively. The dehydration was performed at 4 °C with slight shaking in the dark. Finally, the refractive index was matched by adding dibenzyl ether (DBE) to the 3D GBM spheroids, which were subsequently stored in DBE in airtight glass/PDMS molds in the dark at 4 °C. 3D GBM spheroids were imaged with a confocal microscope (Nikon confocal A1, Nikon Instruments, Tokyo, Japan). Confocal images were analyzed with ImageJ using the “Color Threshold” option to determine the area of the live and dead signal.

### 6.6 GBM spheroid roundness

3D GBM spheroids were generated as previously described and cultured for a total duration of 12 days, while being imaged on various days starting at day 3. The circularity and roundness was determined using ImageJ based on the drawn outline for each spheroid.

### 6.7 Scanning electron microscopy of 3D GBM spheroids

For scanning electron microscopy, 3D GBM spheroids were generated as previously described. After 5 days of culture, 3D GBM spheroids were washed with DPBS before being fixed with 2.5 v/v% glutaraldehyde (Electron Microscopy Sciences, Hatfield, PA, USA) for 1 h at room temperature and at 4 °C overnight. The fixed 3D GBM spheroids were washed three times with MilliQ water before being frozen in liquid nitrogen. Afterwards the 3D GBM spheroids were lyophilized (TFD5503 Freeze Dryer, ilShin BioBase Europe, Ede, The Netherlands), gold-sputtered (Sputter Coater 108 Auto, Cressington Scientific Instruments, Watford, UK) and imaged using a scanning electron microscope (JSM-IT100, JEOL, Tokyo, Japan) at an accelerating voltage of 5 kV and a probe current of 35.

### 6.8 Hematoxylin/Eosin Staining of 3D GBM spheroids

For hematoxylin/eosin (H/E) staining, 3D GBM spheroids were generated, fixed with 4 v/v% formaldehyde after 5 days of culture, embedded in Cryomatrix™ and cryosectioned as previously described. Cryosectioned 3D GBM spheroids were fixed again with 4 v/v% formaldehyde for 15 min, washed twice with MilliQ and incubated with hematoxylin (Sigma-Aldrich) for 15 min before being rinsed under tap water for 15 min. Next, the 3D GBM spheroids were incubated with eosin solution (Sigma-Aldrich) for 90 s before being washed in 96 v/v% ethanol. After washing, the 3D GBM spheroids were dehydrated using a series of 2 × 96 v/v% ethanol and 2 × 100% ethanol (1 min incubation). Dehydrated 3D GBM spheroids were mounted using DPX mounting for histology solution (Thermofisher Scientific) before being imaged using Nanozoomer-RS.

### 6.9 FITC-dextran penetration into 3D GBM spheroids

3D GBM spheroids were generated as previously described. 5 days post-seeding, the 3D GBM spheroids were incubated with fluorescein isothiocyanate (FITC) – dextran (average molecular weight of 150.000 g/mol, Sigma-Aldrich) at a concentration of 100 μg/mL. After 2 days of incubation, the 3D GBM spheroids were washed twice with DPBS before being fixed with 4 v/v% formaldehyde, embedded in Cryomatrix™ and cryosectioned as previously described. The cryosectioned 3D GBM spheroids were air-dried, rehydrated using PBS and immediately mounted with Fluoroshield™ with DAPI before being imaged using Nanozoomer-RS. The images were quantified using ImageJ. Calculated percentage values are determined by calculating the positive area for FITC-Dextran standardized to the total area for the DAPI signal.

### 6.10 GBM spheroid size and growth rate

3D GBM spheroids were generated as previously described and cultured for a total duration of 12 days, while being imaged on various days starting at day 3. The size of the 3D GBM spheroids was determined using ImageJ based on the drawn outline for each spheroid. For the growth rate, the size on the different days was standardized to day 3 as starting day of the imaging.

### 6.11 GBM spheroid migration on Matrigel™

3D GBM spheroids were generate and cultured as previously described. After 5 days of culture, a thin hydrogel layer of growth factor-reduced Matrigel™ (Corning Inc., Corning, NY, USA) of 1–2 mm thickness was formed in custom-made PDMS (Dow Sylgard™ 184 Silicone Elastomer, Mavom BV, Alphen aan den Rijn, The Netherlands) wells. The PDMS wells were generated by punching 3 mm holes into a 2–3 mm thick film of PDMS before binding it to a thin glass slide using plasma cleaning to allow for optical clearance. After a stable Matrigel™ hydrogel was formed, the 3D GBM spheroids were placed onto the hydrogel and the migration was tracked for additional 4 days while being imaged every day using an EVOS M5000 microscope. The images were analyzed using ImageJ. The relative migration is based on the calculated surface area for the migrated cells standardized to day 0 (placement of 3D GBM spheroids onto the hydrogel).

### 6.12 Gene expression profile of 3D GBM spheroids

3D GBM spheroids were generated and cultured as previously described. After 5 days, at least 10 spheroids/conditions were pooled and the total RNA was isolated using the GenElute™ Mammalian Total RNA Miniprep Kit (Sigma-Aldrich) and the RNA amount was measured by a Nanodrop® ND-1000 Spectrophotometer (Thermofisher Scientific). Next, cDNA was synthesized with iScript™ cDNA Synthesis Kit (BioRad, Veenendaal, The Netherlands). For each PCR reaction 10 ng cDNA was used. All real-time PCR primers (Supplementary Table S2) were purchased from Sigma-Aldrich (abbreviation of all different genes in Supplementary Table S1). Quantitative real time PCR was performed with the 2× Sensimix SYBR and Flurescein Kit (Bioline GmBH, Luckenwalde, Germany) using a BioRad CFX384 real-time PCR detection system (BioRad). All gene expression levels were normalized to the expression of the house-keeping gene RPS18.

### 6.13 Incubation of 3D GBM spheroids with Natural Killer (NK92) cells

3D GBM spheroids were generated and cultured as previously described. After 5 days of culture, the GBM spheroid culture medium was completely removed and either 15,000 or 30,000 NK92 cells were added per well in full NK92 cell culture medium. In addition, 3D GBM spheroids were incubated with full NK92 cell culture medium without NK92 cells serving as control. This step was redone 8 days post-seeding completely refreshing the used NK92 cells. The 3D GBM spheroids were imaged on day 3 and day 5–11, while imaged daily during this period. The growth rate was determined by standardizing the size of the spheroids to day 5, the initial day of the incubation with NK92 cells for the first time. Gene expression of relevant immune suppression markers was determined as aforementioned.

### 6.14 Schematics and statistical analysis

All graphs were made using GraphPad Prism Vol.9 (GraphPad Software Inc., San Diego, CA) based on calculations using Microsoft Excel. Schematics were made using Inkscape (Open-source vector graphics editor). All values are expressed as mean ± standard error of the mean (SEM). Statistical significance of the results was performed by either two-tailed unpaired student’s *t*-test for comparison between two treatment groups or two-way ANOVA for comparing multiple treatment groups. Significant difference was determined for a p-value of *p < 0.05, **p < 0.01 and ***p < 0.001, respectively.

## CRediT authorship contribution statement

**Marcel Alexander Heinrich:** Writing – review & editing, Writing – original draft, Methodology, Investigation, Formal analysis, Data curation, Conceptualization. **Ngoc-Tien Huynh:** Methodology, Formal analysis, Data curation. **Lena Heinrich:** Methodology, Formal analysis, Data curation. **Jai Prakash:** Writing – review & editing, Supervision, Resources, Project administration, Funding acquisition, Conceptualization.

## Declaration of competing interest

The authors declare the following financial interests/personal relationships which may be considered as potential competing interests:
