# Supplementary material for: Understanding glioblastoma stromal barriers against NK cell attack using tri-culture 3D spheroid model
Source: Heliyon. 2024 Jan 20;10(3):e24808. doi: 10.1016/j.heliyon.2024.e24808 (PMC10838749; doi:10.1016/j.heliyon.2024.e24808)
Supplement: Multimedia component 1 [file mmc1.docx]

Supporting Information

Understanding glioblastoma stromal barriers against NK cell attack using tri-culture 3D model

Marcel Alexander Heinrich^1^, Ngoc-Tien Huynh^1^, Lena Heinrich, Jai Prakash*

| Table S1. Full name and alternative names for abbreviated genes. | | |
| --- | --- | --- |
| Abbreviation | **Full Name** | **Also known as:** |
| Genes related to treatment resistance and stroma activation | | |
| GFAP | Glial fibrillary acidic protein | ALXDRD |
| POSTN | Periostin | OSF2, OSF-2, PDLPOSTN, PN |
| VIM | Vimentin |  |
| ANXA1 | Annexin A1 | ANX1, LPC1 |
| NES | Nestin | Nbla00170 |
| FGF2 | Fibroblast growth factor 2 | BFGF, FGFB, HBGF-2 |
| CX3CL1 | C-X3-C motif chemokine ligand 1 | ABCD-3, CXC3, NTN, NTT, SCYD1 |
| PDGFRβ | Platelet-derived growth factor receptor beta | CD140B, IBGC4, IMF1, JTK12, KOGS, PENTT |
| CHI3L1 | Chitinase 3 like 1 | ASRT7, GP39, YK40, YKL40, hGCP39 |
| GPNMB | Glycoprotein nmb | HGFIN, NMB, PLCA3 |
| Genes related to NK cell suppression | | |
| IDO1 | Indoleamine 2,3-dioxygenase 1 | IDO, INDO |
| PTGES2 | Prostaglandin E synthase 2 | C9orf15, GBF1, PGES2 |
| 5’-NT (CD73) | 5’-nucleotidase ecto | NTE, NT, CALJA |
| PD1-L1 (CD274) | Programmed cell death 1 ligand 1 | B7-H, PDCD1L1 |
| General housekeeping gene | | |
| RPS18 | Ribosomal protein S18 | D6S218E, HKE3, S18, uS13 |

| Table S2. Sequences of primers used in real-time PCR. | | |
| --- | --- | --- |
| Abbreviation | **Forward Primer** | **Reverse Primer** |
| Genes related to treatment resistance and stroma activation | | |
| GFAP | AGATTGAGTCGCTGGAGGAG | GACGCCATTGCCTCATACTG |
| POSTN | ACAGAGAGGTCACCAAGGTC | CTTGCAACTTCCTCACGGGT |
| VIM | AAATGGCTCGTCACCTTCGT | CAGCTTCCTGTAGGTGGCAA |
| ANXA1 | CACAGCTATCGTGAAGTGCG | ATGGCGAGTTCCAACACCTT |
| NES | ATTTGAGGACCTGGGGACTG | CTCATCTGCAAACCCATCGG |
| FGF2 | GGCTTCTTCCTGCGCATCCA | GCTCTTAGCAGACATTGGAA |
| CX3CL1 | GAGTCTGAAGGCTGGGTTCT | TGGTAAGGACTGTGAGGCTG |
| PDGFRβ | AGGCAAGCTGGTCAAGATCT | GCTGTTGAAGATGCTCTCCG |
| CHI3L1 | CGACCAGGAAAGCGTCAAAA | ATGGCATTGGTGAGAGGGAA |
| GPNMB | TCACGAGCACCCTGATTTCT | CTGACCACATTCCCAGGACT |
| Genes related to NK cell suppression | | |
| IDO1 | TGCAAGAACGGGACACTTTG | CCCTTCATACACCAGACCGT |
| PTGES2 | GACAGCAGGACGGTTTGTTT | TTGCAAACATGTCCCAGTCC |
| 5’-NT (CD73) | ACAACCTGAGACACACGGAT | TAACTGGGCACTCGACACTT |
| PD1-L1 (CD274) | ATTCCGGCAGTGTACCTTGA | CAAGGGTTCAAGCACAACGA |
| General housekeeping gene | | |
| RPS18 | TGAGGTGGAACGTGTGATCA | CCTCTATGGGCCCGAATCTT |


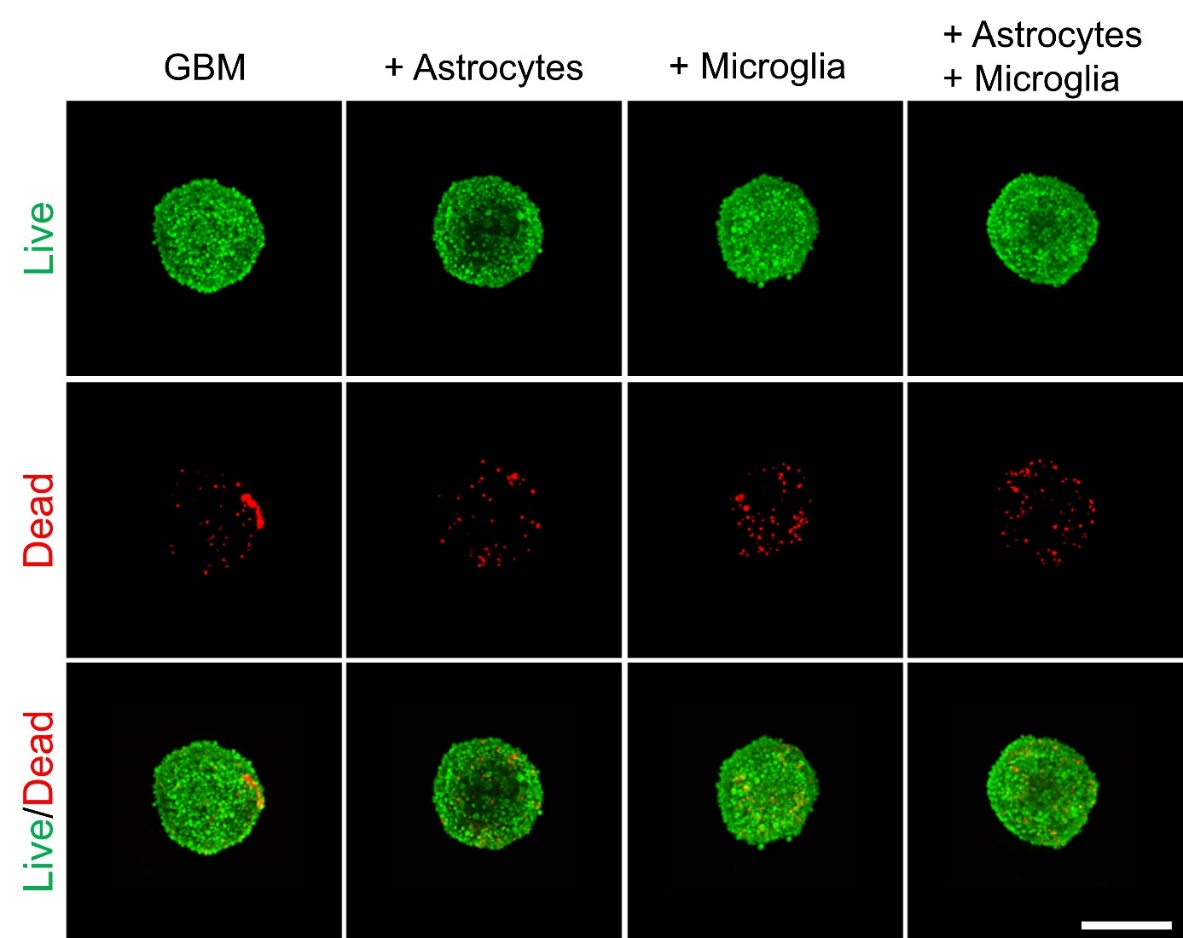


**Figure S1.** LIVE (green)/ DEAD (red) of optically cleared 3D GBM spheroids by confocal microscopy on day 5 post seeding highlighting LIVE, DEAD and merged images (scale = 300µm).


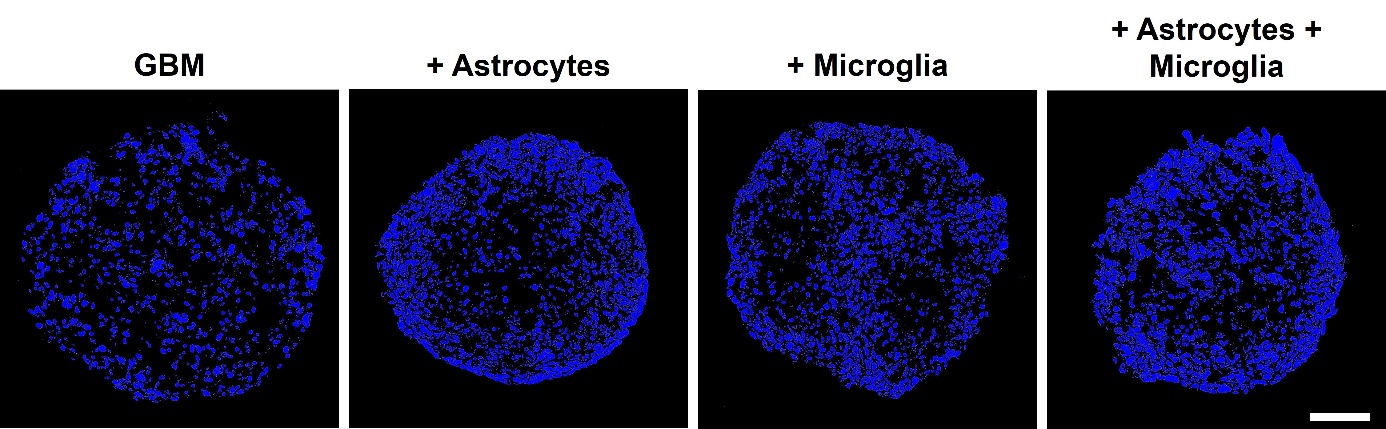


**Figure S2. Cell distribution in gliospheroids**. Map indicating the positioning of nuclei within the 3D GBM spheroids based on the H/E staining in Figure 2E (scale = 100µm).


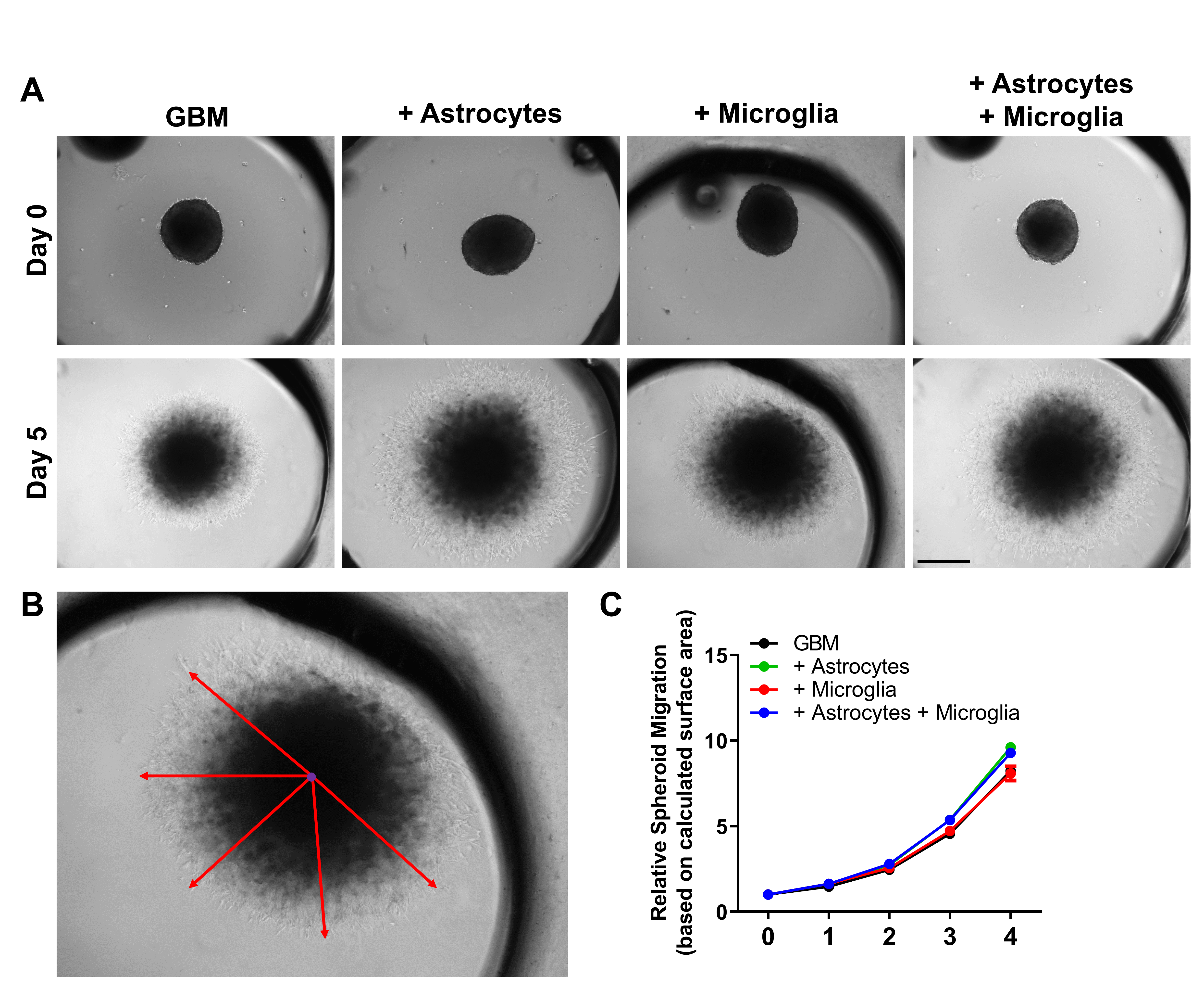


**Figure S3. Migration of gliospheroids.** A) Microscopic images of 3D GBM spheroids on day 0 for migration (+3 days after initiation of culture) and on day 5 (+3), scale = 500 µm. B) Highlighted microscopic image demonstrating the individual measurement for each spheroid. Only the migration towards the center of the well was measured to avoid bias due to migration limited by the well wall. C) Migration curve standardized to day 0, n = 4.


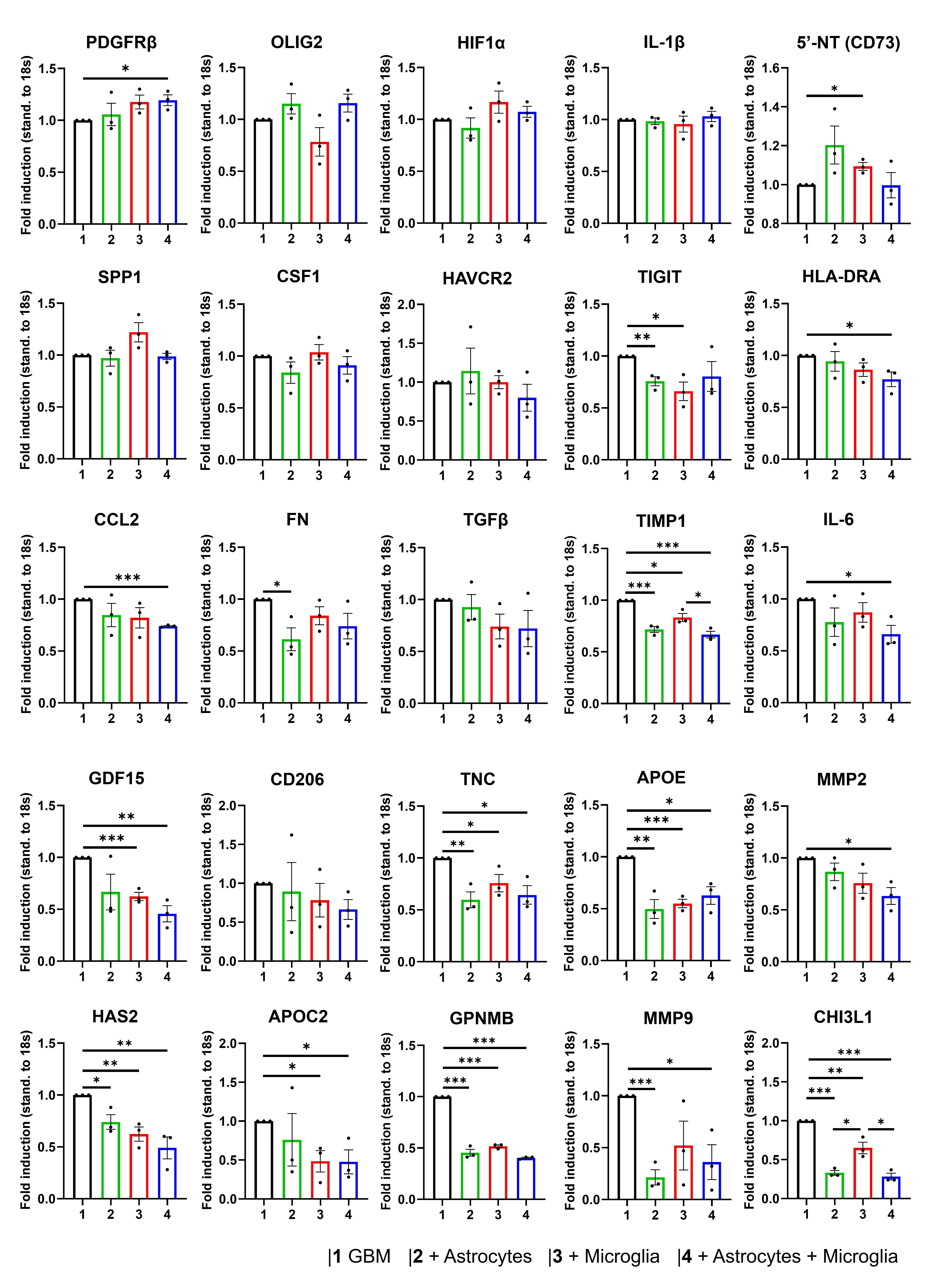


**Figure S4. Gene expression profile of gliospheroids.** Individual expression profiles highlighting comparing GBM, +astrocytes, +microglia and the triculture, n = 3. Data present mean ± standard error of the mean. Statistical analysis was performed by two-tailed Student’s t-test. *p < 0.05, **p < 0.01, ***p < 0.001.

**Figure S5. Treatment of 3D GBM spheroids with 15,000 NK-92 cells.** A) Growth rate of 3D GBM spheroids after the incubation with vehicle or 15,000 NK-92 cells/ well. Growth rate is standardized to day 5 (start of the co-culture) and followed for 6 consecutive days after start of the co-culture. Arrows indicate addition of NK-92 cells, n = 3. B) Microscopic images of 3D GBM spheroids on day 11 incubated with either vehicle (NK-92 cell medium) medium or 15,000 NK-92 cells (scale = 500 µm). C) Highlighted growth rate of 3D GBM spheroids on day 11. Each conditions was standardized to its representative control group treated with vehicle. Data represent mean ± standard error of the mean. Statistical analysis was performed by two-tailed Student’s t-test comparing two specific groups.
